# Genetic Relationship of Brassicaceae Hybrids with Various Resistance to Blackleg Is Disclosed by the Use of Molecular Markers

**DOI:** 10.3390/cimb44090295

**Published:** 2022-09-17

**Authors:** Justyna Szwarc, Janetta Niemann, Joanna Kaczmarek, Jan Bocianowski, Dorota Weigt

**Affiliations:** 1 Department of Genetics and Plant Breeding, Poznań University of Life Sciences, Dojazd 11, 60-632 Poznan, Poland; 2 Institute of Plant Genetics of the Polish Academy of Sciences, Strzeszyńska 34, 60-479 Poznań, Poland; 3 Department of Mathematical and Statistical Methods, Poznań University of Life Sciences, Wojska Polskiego 28, 60-637 Poznań, Poland

**Keywords:** Brassicaceae, interspecific hybrids, SSR markers, *Leptosphaeria maculans*, genetic similarity

## Abstract

*Brassica napus* is an important oil source. Its narrow gene pool can be widened by interspecific hybridization with the Brassicaceae species. One of the agronomically important traits, that can be transferred through the hybridization, is the resistance to blackleg, a dangerous disease mainly caused by *Leptosphaeria maculans*. Hybrid individuals can be analyzed with various molecular markers, including Simple Sequence Repeats (SSR). We investigated the genetic similarity of 32 Brassicaceae hybrids and 19 parental components using SSR markers to reveal their genetic relationship. Furthermore, we compared the field resistance to blackleg of the interspecific progenies. The tested set of 15 SSR markers proved to be useful in revealing the genetic distances in the Brassicaceae hybrids and species. However, genetic similarity of the studied hybrids could not be correlated with the level of field resistance to *L. maculans*. Moreover, our studies confirmed the usefulness of the Brassicaceae hybrids in terms of blackleg management.

## 1. Introduction

Brassicaceae is a family of high agroeconomic importance comprising fodder, oilseed plants, vegetables, ornamental species, as well as plants of medical and scientific importance. Furthermore, ecological, morphological, and genetic diversity of this family makes it a perfect model for relationship and evolution studies [1]. The genus *Brassica* contains three diploid species, i.e., *B. rapa* (AA genome), *B. nigra* (BB genome), and *B. oleracea* (CC genome), and allotetraploid species obtained as a result of natural interspecific crosses, namely *B. napus* (AACC), *B. juncea* (AABB), and *B. carinata* (BBCC). Another representative of the Brassicaceae family is *Sinapis alba*, a yellow mustard plant closely related to *Brassica*, well known for possessing many potentially useful traits [2].

*Brassica napus* (rapeseed) is one of the most important oil crops, accounting for over 12% of worldwide oil production (USDA). Due to a relatively short history of cultivation and use of conventional breeding methods, rapeseed displays limited genetic diversity [3,4]; therefore, it seems crucial to expand the *B. napus* gene pool. One of the most effective approaches to solve this problem is interspecific hybridization [5]. Crossing the rapeseed with different species may help to enrich the *B. napus* germplasm and to enable the transfer of genome fragments carrying desirable traits, which could further improve the cultivar’s characteristics. The sexual incompatibility and differences in the genome sizes of parental components may result in hybridization failure [6]. Barriers of interspecific hybridization can be overcome by implementing in vitro techniques, including ovary, ovule, and embryo rescue [7]. The Department of Genetics and Plant Breeding of Poznań University of Life Sciences has great experience in creating interspecific Brassicaceae hybrids, which are profoundly analyzed in terms of chromosomal constitution, morphology, as well as insect and pathogen resistance. Recently developed hybrids showed a significant variability of blackleg resistance in field conditions. Blackleg, mainly caused by *L. maculans*, is a fungal disease which can cause significant yield losses [8]. The reliance on commercial cultivars with a single resistance source increases the selective pressure on pathogens and accelerates its evolution. Management of blackleg disease includes proper agronomic practices (such as crop rotation and tillage), the use of fungicides, weed control, the use of certified seeds and the use of resistant cultivars [9]. The breeding of resistant cultivars is environmentally friendly and is a reliable method of controlling blackleg disease [10]. It relies on the existence of naturally resistant genotypes, which can be used as a donor of certain genes conferring blackleg resistance.

Various molecular marker systems such as RFLP, SSR, and RAPD can be used to determine the genetic distance of the Brassicaceae species [11]. Simple Sequence Repeats (SSR) or microsatellites are defined as tandem repeats of short nucleotide motifs, usually consisting of 1–6 base pairs [12]. They occur frequently in eucaryotic organisms, and the variation in repeat numbers results in a high degree of polymorphism. The random distribution of SSR loci in plant genomes allows for genetic differentiation within and between species [13]. Moreover, it defines the high utility of SSR markers for cruciferous plants, as the microsatellite loci among members of the Brassicaceae family show high variation in length, which subsequently permits the differentiation of species [14,15]. The SSR markers had been previously used in numerous Brassicaceae studies, including unraveling the genetic variation and species diversity [14,16,17], species and cultivar differentiation [13,18], and estimation of genetic distances [19].

We are aiming to gain insight into the genetic relationship between hybrids with different parental components, which are diverse in terms of resistance to blackleg. Therefore, the objectives of this research are to determine the genetic similarity of hybrid and parental genotypes from the Brassicaceae family and to evaluate the usefulness of the chosen SSR markers for genetic diversity analysis.

## 2. Materials and Methods

A total of 32 various Brassicaceae hybrids of F_9_ and F_10_ generation and 19 parental genotypes were used as research material (Table 1). Interspecific hybrids of the F_1_ generation were developed at the Department of Genetics and Plant Breeding (Poznań University of Life Sciences), with the use of in vitro cultures. Next, selected combinations were self-pollinated multiple times in order to obtain stable hybrid lines.

### 2.1. Molecular Analysis

15 SSR markers were selected to characterize the genetic background of the research material. The markers were chosen according to the literature data [20]. This set of microsatellites was developed from *B. rapa* using the ISSR-suppression-PCR method. Preliminary screening was performed in order to assess their usefulness in the present study. Genomic DNA was extracted from young seedling leaves of the studied individuals using the Genomic Mini AX Plant kit (A&A Biotechnology, Gdańsk, Poland) according to the manufacturer’s protocol. PCR was performed in a total volume of 12.5 µL (6.25 µL OptiTaq Master Mix (EURx, Gdańsk, Poland), 2 × 0.5 µL primers, 4.25 µL H_2_O, and 1 µL DNA template) under the following conditions: initial denaturation at 94 °C for 5 min, thirty-five cycles of amplification (denaturing at 94 °C for 45 s, annealing at primer specific temperature for 45 s, extension at 72 °C for 1.5 min), followed by a final extension step at 72 °C for 7 min. Primer sequences and annealing temperatures are presented in Table 2. 

Electrophoresis was performed on agarose gel stained with Midori Green Advance (Nippon Genetics, Düren, Deutchland), 5 µL per 100 mL of TBE buffer. All image data obtained from the electrophoresis gels were examined in the same way: for each marker, the presence or absence of a band of particular size was scored as ‘1’ or ‘0’, respectively. Next, a binary data matrix was created which was further analyzed with Peak Scanner Software v1.0 (Applied Biosystems, Waltham, MA, USA).

### 2.2. Statistical Analysis

The polymorphic information content (PIC) was calculated for each marker using the formula:

$$PIC_{i}=1-\overset{k}{\underset{j=1}{\sum }}p_{ij}{}^{2},$$

where *p_ij_* denotes frequency of the *j*th allele for *i*-th marker among a total of *k* alleles [21,22].

Genetic similarity (*GS*) was estimated for each pair of genotypes on the basis of Nei and Li [23]:

$$GS=\frac{2N_{AB}}{N_{A}+N_{B}},$$

where *N_AB_* denotes the number of bands in genotypes **A** and **B**, *N_A_* and *N_B_* denote the number of bands in **A** and **B**, respectively. The similarity matrix was used to construct a dendrogram using the unweighted pair group method with arithmetic mean (UPGMA) to determine genetic relationships among the genotypes studied. The principal component analysis (PCA) was calculated on the basis of the similarity matrix. All the analyses were conducted using the GenStat 18.2 edition (VSN International Ltd., Hemel Hempstead, UK) statistical software package. The analysis of molecular variance (AMOVA) was made using GenAlEx 6.5 [24]. AMOVA estimated and partitioned the total molecular variance between and within the groups of genotypes and tested the partitioned variance components [25]. The population genetic structure coefficient (*F_ST_*) was calculated using the formula:

$$F_{ST}=\frac{H_{T}-H_{S}}{H_{T}},$$

where *H_T_* denotes the probability that two alleles drawn at random from the entire group differ in state and *H_S_* denotes the probability that two alleles drawn at random from a subgroup differ in state. Groups for AMOVA, presented in the Table 1, were created by organizing the genotypes on the basis of their parental components, e.g., *B. napus* × *S. alba* hybrids were grouped together with *S. alba*. Four *B. napus* cultivars were added to each group.

### 2.3. Resistance to Blackleg

All hybrid combinations have been studied in terms of resistance to phoma leaf spotting/blackleg in field conditions. The assessment was carried out in testing fields at the Poznań University of Life Sciences experimental station Dłoń, located in Wielkopolska Voivodeship. The soil and weather conditions were typical for this region of Poland, and no fungicides or pesticides were used on the testing field. The agricultural practices were optimal for the local ecological conditions. The experiment was set up in a completely randomized block design with five replications; the size of a single plot was 10 m^2^ with a 0.30 m row distance and a sowing density of 60 seeds per square meter. The assessment was performed in two terms, i.e., in November, BBCH 19 (term I), and July, BBCH 70-89 (term II). Phoma leaf spotting (term I) was evaluated according to the scale from 0 to 4, where 0 was no visible disease symptoms and 4 was numerous (over 10) leaf spots per plant [26]. The blackleg symptoms (term II) were assessed according to a scale from 0 to 9, where 0 was no visible symptoms and 9 was a plant totally damaged by the disease [26]. Obtained scale values were subsequently transformed into percentage values. For every genotype, 10 randomly chosen individuals were examined, and for each genotype, the average values from 10 replications were calculated.

## 3. Results

### 3.1. Genetic Similarity Assessment

**Figure 1:**
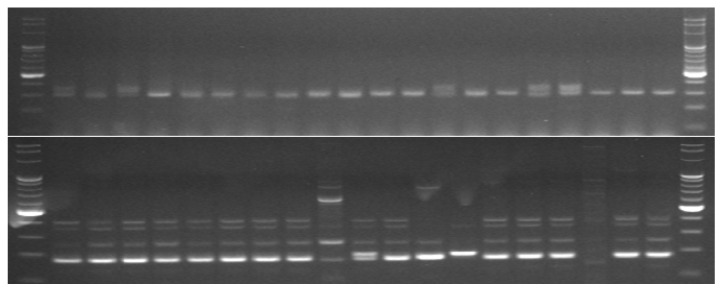
Example of electrophorograms with visible PCR products. Results for genotypes 21–40, marker mstg004 (**above**) and mstg008 (**below**).

The set of 15 primer pairs allowed for the detection of 2 monomorphic and 98 polymorphic alleles (Table 3, Figure 1). The average number of polymorphic alleles per marker was 6.533, ranging from 2 to 15. Monomorphic alleles were observed only for two markers: mstg028 and mstg042. The SSR markers used in this study generated highly informative loci with the PIC values ranging from 0.594 for mstg016 to 0.989 for mstg039, with the mean 0.848 (Table 3). 

**Figure 2:**
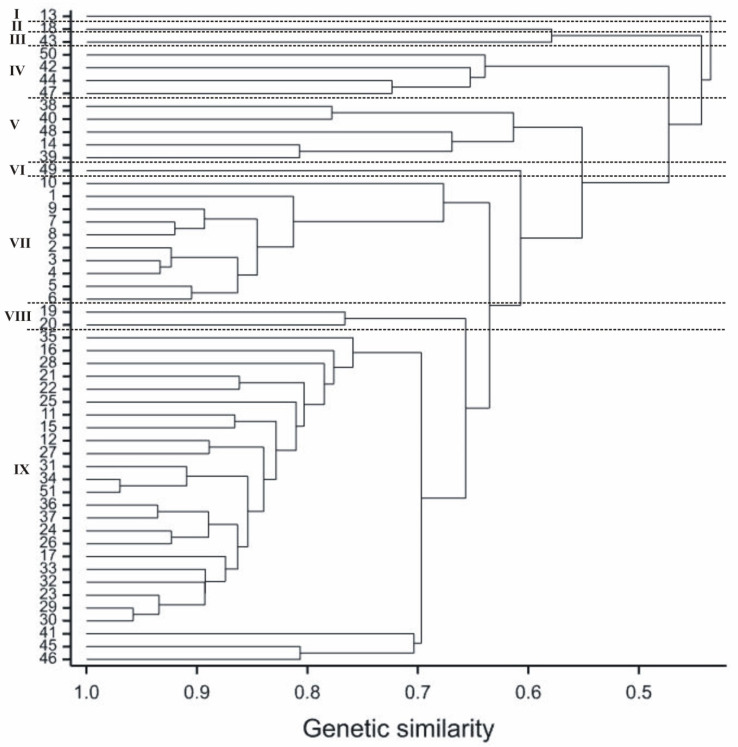
Dendrogram obtained from SSR data showing the genetic relationship of studied genotypes (numbers according to Table 1). Genotypes were grouped hierarchically using the UPGMA method. The scale at the bottom of the dendrogram indicates the level of similarity between individual plants.

The data were computed to estimate genetic similarity between the studied rapeseed genotypes based on Nei and Li’s coefficients. The highest genetic similarity (equal to 0.97) was found between genotypes *B. napus* cv. Zhongshuang9 × *B. rapa* ssp. *pekinensis* 08 006169 (34) and *B. napus* cv. Zhongshuang9 × *B. rapa* ssp. *pekinensis* 08 006169 (51), whereas the lowest genetic similarity (0.22) was found for *B. carinata* (7) and *B. fruticulosa* PI 649097 (18). The mean value of genetic similarity was 0.63. The SSR marker data were used to group cultivars by the UPGMA method. The relationships between genotypes are presented in the form of a dendrogram (Figure 2), in which nine clusters were clearly distinguished. Cluster I comprised only one individual, genotype 13 (*B. rapa* ssp. *chinensis* (COBORU)), which had less than a 0.5 similarity with other genotypes; Cluster II comprised only one individual, genotype 18 (*B. fruticulosa* PI 649097); Cluster III comprised only one individual, genotype 43 (*B. napus* cv. Lisek × *B. fruticulosa*—PI649099); Cluster IV comprised genotypes 42, 44, 47, and 50 (*B. napus* cv. Jet Neuf × *S. alba* cv. Bamberka, *B. napus* cv. Lisek × *S. alba* cv. Bamberka, *B. napus* cv. Californium × *S. alba* cv. Bamberka, and *S. alba* cv. Bamberka); Cluster V, 14, 38, 39, 40, and 48 (*B. rapa* ssp. *chinensis* PI430485 98CI, *B. rapa* ssp. *pekinensis* 08, 007569, *B. rapa* ssp. *pekinensis* 08, 007574, *B. rapa* ssp. *pekinensis* (COBORU), and *B. rapa* ssp. *pekinensis* 08 006169); Cluster VI comprised only one individual, genotype 49 (*B. oleracea* var. *alboglabra*); Cluster VII comprised genotypes 1, 2, 3, 4, 5, 6, 7, 8, 9, and 10 (*B. napus* cv. Jet Neuf × *B. carinata* PI 649091, *B. napus* cv. Lisek × *B. carinata* Dodola, *B. napus* cv. Jet Neuf × *B. carinata*—PI 649094, *B. napus* cv. Jet Neuf × *B. carinata*—PI 649096, *B. carinata* 1, *B. carinata* 2, *B. carinata* 3, *B. carinata* 4, *B. carinata* cv. Dodola, and *B. carinata* PI 596534); Cluster VIII comprised two genotypes, 19 and 20 (*B. napus* cv. Californium × *B. fruticulosa*—PI649097 and *B. napus* cv. Lisek × *B. fruticulosa*—PI649097), while the ninth cluster contained the remaining 26 genotypes (Figure 2).

The significant differentiation (*F_ST_* = 0.059; *p* = 0.011) between the genotypes among the groups presented in Table 1 was further supported by the AMOVA results. The intra- and inter-genotype variabilities were found to be significant, with 6% of the genetic variance contributed by the differentiation between the groups, whereas 94% was partitioned within the groups. The largest variability was observed in the first group (mean squares within the group was equal to 9.582), while the smallest was in group number 7 (4.160) (Table 4).

Statistical significant differences were observed between the following pairs of groups of genotypes: 1–2, 1–3, 1–4, 1–5, 1–6, 3–4, 3–5, and 4–6 (Table 4).

**Figure 3:**
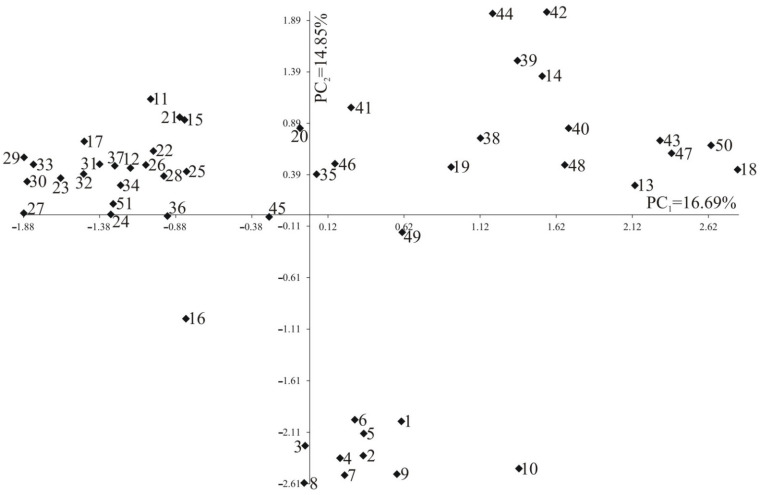
Principal component analysis of 51 genotypes based on 100 detected PCR products, numbers 1–51 according to Table 1.

The PCA for 51 genotypes based on the distance matrix was presented in Figure 3. The first two PCs explained a total of 31.54% SSR marker variation (16.69% and 14.85%, respectively).

### 3.2. Field Resistance to Blackleg

**Figure 4:**
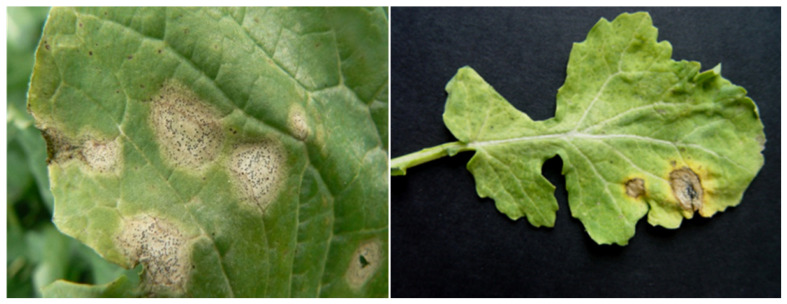
Examples of leaf damage on hybrid genotypes caused by *L. maculans*.

The performed analysis allowed to distinguish the genotypes with the highest resistance level to blackleg (Table 5). Sixteen hybrid combinations belonged to the statistically best group (group f) in both terms, which indicates their ability to maintain stable and low susceptibility to pathogen infestation. These include hybrids with *B. carinata*, *B. fruticulosa*, and *S. alba* as a parental component. The lowest level of blackleg resistance was observed for *B. napus* cv. Górczański × *B. rapa* ssp. *chinensis* in both terms (23.33% and 25% infestation), although those genotypes are still considered as moderately resistant. Examples of lesions observed on hybrid combinations are presented in Figure 4.

## 4. Discussion

The assessment of diversity between species is important for the management of germplasm resources and for the curation of genetic databases. As the phenotypic assessments partially relay on environmental conditions, they do not allow for a clear discrimination of related species. Thus, in this study, genotypic analysis using SSR markers was performed for the unbiased determination of genetic diversity.

Molecular DNA markers are important tools for genetic similarity studies. SSR markers are especially valuable, as they enable multi-allelic detection and can be applied using various laboratory systems [27]. The markers selected for this study derived only from *B. rapa* (AA, 2n = 20) and were developed using the ISSR-suppression-PCR method by Tamura et al., [20]; however, the applicability of these markers for a wider group of *Brassica* species has been suggested by the aforementioned authors. The Brassicaceae family consists of approximately 3000 species [28] with diverse genomic composition, e.g., the U triangle (A, B, and C genome), *S. alba* (S genome), and *B. fruticulosa* (F genome), although conserved regions of gene content and gene order are present among the family [29]. This attribute, combined with the before mentioned unique features of the microsatellite loci that are widely spread among the Brassicaceae, allows to detect sequences originating from one species in the genomes of its relatives. We managed to confirm that the selected SSR markers can be used for genetic similarity studies in the Brassicaceae family, as the markers enabled the detection of allelic variation. 

Polymorphism Information Content (PIC) is an indicator that allows to evaluate the discriminatory ability of molecular markers and to study the genetic diversity [30]. The PIC value can vary from 0 to 1, and markers with a PIC value exceeding 0.7 are considered highly informative [31]. Therefore, it can be concluded that twelve out of fifteen tested markers are particularly effective in detecting the polymorphism in the studied population.

The UPGMA allowed for the distinction of nine groups, based on genetic similarity. Generally, the applied method permitted the assessment of the genetic distance of the studied hybrids and their parents, but not all of the results are in line with the predictions. For example, *B. rapa* ssp. *chinensis* (COBORU) shows weak connection to their progeny or other genotypes form the same species. Furthermore, the distinctiveness of this genotype was confirmed with the PCA method. The weaker-than-expected association between species can be explained by a different origin (geographical distribution) or outbreeding [32].

The PCA analysis was conducted to confirm the complicated structure of the studied individuals, and the results confirmed a close relationship for *B. rapa* and *B. carinata* and their hybrid progeny. The rest of the genotypes were generally more scattered around the diagram. However, attention should be drawn to the short distance revealed for two pairs of genotypes: *B. napus* cv. Lisek × *B. fruticulosa* PI649099 and *B. napus* cv. Californium × *S. alba* cv. Bamberka, and *B. napus* MS8 line × *B. rapa* ssp. *pekinensis* 08 006169 and *B. napus* cv. Jet Neuf × *B. oleracea* var. *alboglabra*. These hybrids’ male parental components present entirely different genomic structures, however their genetic similarity can be explained by the unequal inheritance of the *B. napus* genome during hybridization. It should also be emphasized that the markers used in this study derived from *B. rapa,* which possess A genome, which might have an impact on the obtained PCR products.

The genetic similarity of the studied genotypes varied from 0.22 to 0.97. The extensive range of the similarity coefficient values show that the Brassicaceae germplasm collection reflects a diverse and varied population. These results are in line with the findings of Kumari et al. [33], as well as other researchers [34], who studied the genetic diversity in nine genotypes of *Brassica* and their wild relatives. 

The level of field resistance to blackleg varied between the studied genotypes. We managed to select sixteen combinations with the lowest pathogen infestation, which might be especially valuable in future studies focusing on finding a durable resistance to *L. maculans* and incorporating their germplasm into the *B. napus* gene pool. All individuals that had *B. carinata*, *B. fruticulosa*, and *S. alba* as one of the interspecific cross components showed the lowest infestation level. This indicates that particular attention should be paid to these parental species, as they may hold valuable resistance genes that could help to control the disease. This is especially important considering the previously reported resistance breakdowns [35]. The aforementioned species have been previously characterized as potentially significant resistance gene sources [36,37,38], which is in line with our findings.

Hybrid individuals with the lowest blackleg infestation could be found in five out of nine groups distinguished with UPGMA and were spread evenly on the PCA diagram. This indicates that the genetic similarity of the studied hybrid genotypes is not correlated with their level of field resistance. On the other hand, it might be simply explained by the fact that applied molecular markers are not linked to the regions of the genome containing the resistance genes.

In conclusion, the tested SSR markers proved to be useful in revealing the genetic distances in Brassicaceae hybrids and species. The ability to properly characterize and organize the genetic resources is key to the effective conservation of accessions. More precise and quick determination of the relationship of genotypes and the amount of variation within or among accessions in a collection can be accomplished by using molecular diagnostic techniques. Other than successfully maintaining the collections, genetic markers are invaluable for crop improvement and plant breeding programs. Moreover, our studies confirmed the usefulness of the Brassicaceae hybrids in terms of blackleg management and the importance of searching new sources of *L. maculans* resistance outside the *B. napus* gene pool.

## Figures and Tables

**Table 1:** List of Brassicaceae genotypes used in this study and groups for the analysis of molecular variance (AMOVA).

No of Genotype	Combination/Species	Group
1	*B. napus* cv. Jet Neuf × *B. carinata* PI 649091	1
2	*B. napus* cv. Lisek × *B. carinata* Dodola	1
3	*B. napus* cv. Jet Neuf × *B. carinata*—PI 649094	1
4	*B. napus* cv. Jet Neuf × *B. carinata*—PI 649096	1
5	*B. carinata* 1	1
6	*B. carinata* 2	1
7	*B. carinata* 3	1
8	*B. carinata* 4	1
9	*B. carinata* cv. Dodola	1
10	*B. carinata* PI 596534	1
11	*B. napus* cv. Górczański × *B. rapa* ssp. *chinensis*	2
12	*B. napus* cv. Zhongshuang9 × *B. rapa* ssp. *chinensis* 08 007574	2
13	*B. rapa* ssp. *chinensis* (COBORU)	2
14	*B. rapa* ssp. *chinensis* PI430485 98CI	2
15	*B. napus* cv. Lisek × *B. rapa* Pak Choi 08, 007574	2
16	*B. napus* cv. Lisek × *B. rapa* Pak Choi 08, 007569	2
17	*B. napus* cv. Górczański × *B. rapa* Pak Choi 08, 007574	2
18	*B. fruticulosa* PI 649097	3
19	*B. napus* cv. Californium × *B. fruticulosa*—PI649097	3
20	*B. napus* cv. Lisek × *B. fruticulosa*—PI649097	3
21	*B.napus* cv. Anderson	1, 2, 3, 4, 5, 6, 7
22	*B. napus* cv. Monolit	1, 2, 3, 4, 5, 6, 7
23	*B. napus* cv. Skrzeszowicki	1, 2, 3, 4, 5, 6, 7
24	*B. napus* cv. Lisek	1, 2, 3, 4, 5, 6, 7
25	*B. napus* cv. Californium × *B. oleracea* var. *alboglabra*	4
26	*B. napus* cv. Jet Neuf × *B. rapa* ssp. *pekinensis* 08 007569	5
27	*B. napus* cv. Jet Neuf × *B. rapa* ssp. *pekinensis* 08 007574	5
28	*B. napus* cv. Górczański × *B. rapa* ssp. *pekinensis* 08.007574	5
29	*B. napus* cv. Górczański × *B. rapa* ssp. *pekinensis* 08.007569	5
30	*B. napus* cv. Californium × *B. rapa* ssp. *pekinensis* 08 007574	5
31	*B. napus* cv. Californium × *B. rapa* ssp. *pekinensis* 08 007574-1	5
32	*B. napus* cv. Californium × *B. rapa* ssp. *pekinensis* 08 007574-2	5
33	*B. napus* cv. Californium × *B. rapa* ssp. *pekinensis* 08 007574-3	5
34	*B. napus* cv. Zhongshuang9 × *B. rapa* ssp. *pekinensis* 08 006169	5
35	*B. napus* MS8 line × *B. rapa* ssp. *pekinensis* 08 006169	5
36	*B. napus* MS8 line × *B. rapa* ssp. *pekinensis* 08 006169	5
37	*B. napus* MS8 line × *B. rapa* ssp. *pekinensis* 08 006169	5
38	*B. rapa* ssp. *pekinensis* 08, 007569	5
39	*B. rapa* ssp. *pekinensis* 08, 007574	5
40	*B. rapa* ssp. *pekinensis* (COBORU)	5
41	*B. napus* cv. Lisek × *B. oleracea* var. *alboglabra*	4
42	*B. napus* cv. Jet Neuf × *S. alba* cv. Bamberka	6
43	*B. napus* cv. Lisek × *B. fruticulosa*—PI649099	3
44	*B. napus* cv. Lisek × *S. alba* cv. Bamberka	6
45	*B. napus* cv. Lisek × *B. tournefortii*	7
46	*B. napus* cv. Jet Neuf × *B. oleracea* var. *alboglabra*	4
47	*B. napus* cv. Californium × *S. alba* cv. Bamberka	6
48	*B. rapa* ssp. *pekinensis* 08 006169	5
49	*B. oleracea* var. *alboglabra*	4
50	*S. alba* cv. Bamberka	6
51	*B. napus* cv. Zhongshuang9 × *B. rapa* ssp. *pekinensis* 08 006169 2	5

**Table 2:** Primer sequences and annealing temperatures of SSR markers used in the study.

SSR Marker	Primer Sequences	Annealing Temperature
mstg001	F: CAT GAG TTT TCA TAA ATA AAA	41 °C
	R: TAT GCA ACT TGT CTT TGA TAT	
mstg004	F: CAT ATA TAG CAT GAG TGG TGC	47 °C
	R: CTT AAA GGG CAC TCT TTC ATG	
mstg008	F: TCT CTT TGA AAT CTC AAC CCA	47 °C
	R: AGA TGG CAT GTT AAA CTG AAC	
mstg012	F: TGA TAC ATA GAC TTG GTG GTG	48 °C
	R: CGG CAT TAT CTT GAA CAC GTT	
mstg013	F: AGA TTT GGC TTA CAC GAC GAC	50 °C
	R: ATA TAC CAG GTA CCG TCA CTC	
mstg016	F: CGT TAC ATT CGG GTA TCA CTA	48 °C
	R: TCA TCG AAA GCC TTG TAA CTG	
mstg025	F: AGA GGC AGT TAC GTT CAC GTC	52 °C
	R: CAT CGC ACT CGT GTC TCT TTC	
mstg027	F: CTC TTT TGG TCA GCT TCC TCA	48 °C
	R: TTG TTA GTT AGA TCC TCG TAG	
mstg028	F: GCC AAG AAG ACG AAG ATT CTC	49 °C
	R: AGG TTC TCG ATT TAG GAA CCG	
mstg033	F: ATG TAA GCA TCT TTG ATC TGC	46 °C
	R: CTT GAT CTT CCT GAT GTA CTC	
mstg034	F: CGA CTG GTA ATA TTC TGA TAC	46 °C
	R: CAT GAA AGA CTC TCA AAT CCC	
mstg038	F: GAA TGG TGG TTC TTG TGT GTC	49 °C
	R: CAA AGC GAA GCT CTT GAA TTG	
mstg039	F: TAC TCG CTC TTG TTG AAG CTG	50 °C
	R: GAC AAT CTT GGA GTC ATC TCG	
mstg042	F: GAT ATT CGA TCC GCT TCG ACA	49 °C
	R: CGA ATA TCT CAT CCA CTT TGT	
mstg052	F: AGT AAC ATG TTT TCT TTT GTG	46 °C
	R: CAT CAG ATG CTC AAG GAA CTT	
mstg055	F: ACA CGC GCC TAT GCA GAA TAC	52 °C
	R: CTT AGC GAT TAC GGT GAA GCC	

**Table 3:** Quantity of detected alleles and PIC values for SSR markers.

SSR Marker	Quantity of Polymorphic Alleles	Quantity of Monomorphic Alleles	Percentage of Polymorphic Alleles (%)	PIC (Polymorphism Information Content)
mstg004	2	0	100	0.962
mstg008	8	0	100	0.969
mstg012	7	0	100	0.771
mstg016	8	0	100	0.594
mstg025	4	0	100	0.838
mstg028	7	1	87.5	0.769
mstg033	3	0	100	0.988
mstg038	9	0	100	0.841
mstg039	15	0	100	0.989
mstg042	2	1	66.7	0.913
mstg052	7	0	100	0.893
mstg055	9	0	100	0.776
mstg001	4	0	100	0.908
mstg034	5	0	100	0.686
mstg027	8	0	100	0.822
Mean	6.533	0.133	96.947	0.848

**Table 4:** Values of differentiation *F_ST_* (below diagonal) and probability based on non-parametric permutational testing procedures with 999 permutations (above diagonal) between groups of genotypes.

Group	1	2	3	4	5	6	7
1	0.000	0.045	0.002	0.016	0.005	0.002	0.055
2	0.041 *	0.000	0.153	0.072	0.421	0.398	0.433
3	0.154 **	0.028	0.000	0.001	0.012	0.052	0.181
4	0.077 *	0.052	0.191 ***	0.000	0.083	0.009	0.060
5	0.066 **	0.000	0.092 *	0.050	0.000	0.289	0.393
6	0.135 **	0.000	0.103	0.160 *	0.017	0.000	0.384
7	0.077	0.000	0.046	0.099	0.000	0.000	0.000
Mean squares within group	9.582	8.132	4.281	8.797	8.853	4.250	4.160

* *p* < 0.05, ** *p* < 0.01, *** *p* < 0.001

**Table 5:** Results of blackleg field resistance assessment for hybrid plants. The level of infestation is expressed as a percentage.

No of Genotype	Combination	Infestation Level—Term I	Infestation Level—Term II
1	*B. napus* cv. Jet Neuf × *B. carinata* PI 649091	0 f *	3 ef
2	*B. napus* cv. Lisek × *B. carinata* Dodola	0 f	3 ef
3	*B. napus* cv. Jet Neuf × *B. carinata*—PI 649094	0 f	4 ef
4	*B. napus* cv. Jet Neuf × *B. carinata*—PI 649096	0 f	3 ef
11	*B. napus* cv. Górczański × *B. rapa* ssp. *chinensis*	23.33 a	25 a
12	*B. napus* cv. Zhongshuang9 × *B. rapa* ssp. *chinensis* 08 007574	15 b	22 ab
15	*B. napus* cv. Lisek × *B. rapa* Pak Choi 08, 007574	8 bcde	8 def
16	*B. napus* cv. Lisek × *B. rapa* Pak Choi 08, 007569	8 bcde	9 cdef
17	*B. napus* cv. Górczański × *B. rapa* Pak Choi 08, 007574	7 cdef	8 def
19	*B. napus* cv. Californium × *B. fruticulosa*—PI649097	0 f	4 ef
20	*B. napus* cv. Lisek × *B. fruticulosa*—PI649097	0 f	5 ef
25	*B. napus* cv. Californium × *B. oleracea* var. *alboglabra*	9.33 bcde	2.08 f
26	*B. napus* cv. Jet Neuf × *B. rapa* ssp. *pekinensis* 08 007569	8 bcde	8 def
27	*B. napus* cv. Jet Neuf × *B. rapa* ssp. *pekinensis* 08 007574	5 def	6 ef
28	*B. napus* cv. Górczański × *B. rapa* ssp. *pekinensis* 08.007574	12.33 bc	15 bcd
29	*B. napus* cv. Górczański × *B. rapa* ssp. *pekinensis* 08.007569	11 bcd	6 ef
30	*B. napus* cv. Californium × *B. rapa* ssp. *pekinensis* 08 007574	5 def	15 bcd
31	*B. napus* cv. Californium × *B. rapa* ssp. *pekinensis* 08 007574-1	4 def	16 bc
32	*B. napus* cv. Californium × *B. rapa* ssp. *pekinensis* 08 007574-2	5.25 def	13.33 cd
33	*B. napus* cv. Californium × *B. rapa* ssp. *pekinensis* 08 007574-3	6 cdef	14 cd
34	*B. napus* cv. Zhongshuang9 × *B. rapa* ssp. *pekinensis* 08 006169	3.33 ef	9 cdef
35	*B. napus* MS8 line × *B. rapa* ssp. *pekinensis* 08 006169 1	4 def	6 ef
36	*B. napus* MS8 line × *B. rapa* ssp. *pekinensis* 08 006169 2	6 cdef	6 ef
37	*B. napus* MS8 line × *B. rapa* ssp. *pekinensis* 08 006169 3	6 cdef	6 ef
41	*B. napus* cv. Lisek × *B. oleracea* var. *alboglabra*	10 bcde	10 cde
42	*B. napus* cv. Jet Neuf × *S. alba* cv. Bamberka	0 f	3 ef
43	*B. napus* cv. Lisek × *B. fruticulosa*—PI649099	0 f	5 ef
44	*B. napus* cv. Lisek × *S. alba* cv. Bamberka	4 def	4 ef
45	*B. napus* cv. Lisek × *B. tournefortii*	8 bcde	6 ef
46	*B. napus* cv. Jet Neuf × *B. oleracea* var. *alboglabra*	10.33 bcde	10 cde
47	*B. napus* cv. Californium × *S. alba* cv. Bamberka	0 f	3 ef
51	*B. napus* cv. Zhongshuang9 × *B. rapa* ssp. *pekinensis* 08 006169 2	6 cdef	15 bcd

* Values with different letters in columns are significantly different.

## Data Availability

Not applicable.
